# Theridion-Curassavicum

**Published:** 1889-03

**Authors:** Daniel W. Clausen

**Affiliations:** Auburn, N. Y.


### THERIDION-CURASSAVICUM.

DANIEL W. CLAUSEN, M. D., *lately of Auburn, N. Y.*

(Read before The Lippe Society, Philadelphia, Pa., Tuesday evening, February 12th, 1889.)

MR. PRESIDENT AND GENTLEMEN:—I hold in my hand a paper which was very hurriedly written and intended to be read before the late Hahnemannian Association of Pennsylvania. On the occasion for which the paper was intended, however, the members of the Association failed to convene—and here lieth the paper unread to any one (save myself), unrevised, and with the same imperfections that covered it when written. Abiding under the shadow of your patience and indulgence, I shall read it as it appeared for the above-mentioned occasion:

*Mr. President and Gentlemen of The Hahnemannian Association of Pennsylvania:*

The present occasion brings me to an outlet of a strait or narrow passage in which I have been confined for several days past. Bound on the one hand by a sense of responsibility for a kindly requested paper as well as by a due appreciation of that request, and on the other hand by a feeling that I could not, under circumstances of domestic affliction, write a paper worthy of your esteem, I have at length concluded to occupy a small space on the broad back of that beautiful spider known as "*Theridion-curassavicum*," and allow it to waft me about the broad field of your wisdom and discussion that I may learn better things concerning the therapeutic value of this beautiful creature that I propose to ride—not as a "hobby," however.

Theridion-curassavicum is a small spider of the West Indies, well known among the people as very poisonous. It is found chiefly on the island of Curaçoa, and frequently among the orange trees, from which fact it is called the "orange spider." "It may be found also in South America. The Arrowackians—the principal tribe that occupied the whole coast from the Oronoko to the Amazon—describe and call it 'Barra garru.'" There has been some dispute, however, regarding the propriety of the name "Theridion," but it is retained as a sufficient identification until the matter is settled by a full scientific description.

As a therapeutic agent, Theridion-curassavicum is mentioned in Stapf's *Archives*, vol. XIV (1834), Jahr's *Symptomen Codex*, Allen's *Encyclopædia of Materia Medica*, in vol. III of the *Volksblätter* (1838), and in a German work where several cures of sheep are recorded (1843), but the best and most complete pathogenesis of the drug is to be found in Hering's *Materia Medica*, vol. I, wherein are contained some well-verified provings by the author on himself and others in 1832. The most remarkable and important circumstances corroborating all the characteristic symptoms have been contributed by Dr. Neidhard, of Philadelphia.

On the mind, and more especially on the sensorium, Theridion acts profoundly. The nerves of sight and hearing are in a high degree of exaltation, and are so sensitive as to be the media through which the sensorium is affected.

MIND.—To the mind of the patient time seems to pass more rapidly. Many provers experienced a great inclination to be startled. There is great aversion to work, even to professional labor.

SENSORIUM.—I would particularly call your attention to the profound vertigo, its conditions and peculiarities of aggravation as found under Therid. The vertigo is renewed by the least motion; increased by every noise or sound (sensitiveness to noise is a strong characteristic of Therid. [Farrington]), and one especial feature of its aggravation from sound lies in the fact that every penetrating sound and reverberation penetrates the whole body, particularly through the teeth, and increases the vertigo, which then causes nausea. Here we perceive a decided action of the drug on the sympathetic nerve. The jar of persons walking the floor, the motion of a vessel, riding in a carriage—these are among the aggravations of Theridion vertigo associated with nausea—deathly nausea and cold sweat. Let me just here suggest that if any of you should ever again have to prescribe for sea-sickness, or for that sickness to which some persons are subject when riding on the cars, please give Theridion a place in the cerebral lobe of your reflection when you are considering the stereotyped Cocculus and Petroleum. A bottle of Theridion (high) should be in the pocket of every homœopath when he is on board a ship, or on the cars. In the intoxication of beginning tobacco smokers this remedy ought to work "like a charm," and probably in certain cases of cholera, where the profound nervous depression exists without the bowel evacuations, it would prove a useful agent.

But there is still another peculiar aggravation of Therid. vertigo: it is on closing the eyes (Thuja); vertigo and nausea when closing the eyes from weariness. Vertigo from stooping also (compare Bell., Calc-carb., Lyc., and many others). Alumina may be *contrasted* with Therid., in some of its mental and sensorial symptoms.

HEAD.—A word now on the headache of Therid. This also is aggravated by motion. Headache behind the eyes (compare Badiaga, Daphne-ind., and Lach.). Head feels very thick, as if it were another, strange head, or belonged to somebody else. Very thick in the head, with nausea and vomiting on the least motion, more particularly when closing the eyes.

TEETH.—Through the teeth Therid. exercises some influence, as I have already shown. Ordinary cold water taken into the mouth affects the teeth as if it were too cold. Every sound penetrates the teeth, *e. g.*, the crowing of cocks.

Please remember the peculiar conditions of aggravation in symptoms of the head, sensorium, and sympathetic stomach troubles, viz.: agg. from noise, every penetrating sound, the jar of persons walking the floor, closing the eyes, motion of the vessel, and riding in a carriage. These are worthy of a place in your memory.

Moschus antidotes the nausea produced by Therid.; Aconite, the sensitiveness to noise and the violent paroxysms.

STOMACH.—Among the symptoms of the stomach we notice qualmishness and vomiting in the morning. Let us remember this in the "morning sickness" of pregnancy. Case.—A woman having had (in child-bed) a violent spell of sickness at the end of the first week, and apparently recovered, was, in the third week, after washing clothes, suddenly attacked with *nausea and fainting;* after it, very pale, and sick at the stomach as soon as she closed her eyes, with vanishing of her thoughts. Smelling of Therid.$^{30}$, she completely recovered (Hering's *Materia Medica*).

NERVES.—From the foregoing statements, we must not wonder that Therid. should exercise a powerful influence on the nervous system generally. It seems analagous to Moschus in certain nervous affections. Hysteria at puberty and at the climaxis. The patient faints after every exertion. Tetanus after the bite of the spider has been observed by Dr. Hille. (There are stinging pains in various parts of the body, and a continuous aching in the left chest, near articulation of floating ribs. [*California Homœopath*, August, 1888.])

HYPOCHONDRIA.—Violent burning pain in the hepatic region, which grows more painful when touched. In abscess of the liver, Dr. Lippe highly recommends it for the relief of the vertigo and nausea. During the pains, retching, vomiting, and bringing up of bile. It has proven a wonderful remedy in the anthrax of sheep, with great tumefaction of the hypogastrium. It cured all cases where the swelling had not turned blue. (When the swelling turns blue, think of Lach., Ars., and Carbo-veg.) The violent burning pain in the hepatic region, other symptoms considered, may be a fine indication for Therid. in cancer of the liver; and in this symptom, Tarentula-cubensis may be favorably compared. For a knowledge of this comparison, I am indebted to my learned friend, Professor J. T. Kent, who has administered Tarentula with remarkable success, when the severe burning pain in cancer of the liver could not be controlled by any other remedy given. A most noted case of the kind was that of a lady, the daughter of a late homœopathic veteran of this city, who never, until death, ceased to thank Professor Kent for the prescription (Tarentula) that had produced so wonderfully soothing an effect upon his daughter, and had rendered her death a painless one.

But, gentlemen, in these few considerations we have, by no means, exhausted all that may be discussed regarding the therapeutic value of Theridion; for, behold! even in that monster—"scrofula"—this valuable drug has been considered indispensable; and Dr. Baruch, of New York, confirmed the supposition contained in the *Archives* of 1834, that Therid. will often be found useful after Calc. and Lyc. have improved the case, but not finished the cure. I will now read an extract of a letter from Dr. Baruch to Dr. C. Hering:

"In cases of scrofulosis, where the best chosen remedies do nothing, I always interpolate a dose of Therid., which must act for eight (8) days; and I have seen the most surprising results from it, particularly in caries and necrosis. For phthisis Florida, Therid. is indispensable, and effects an entire cure if given in the beginning of the disease. In cases of rachitis, caries, and necrosis, I depend chiefly on Therid., which, although it does not seem to affect the external scrofulous symptoms, apparently goes to the root of the evil, and effectually destroys the cause of the disease."

Now, gentlemen, shall we dare presume Theridion-cur. to be an antipsoric remedy? Let us study it. The contents of this paper are by no means exhaustive; they are merely suggestive; but if they shall move any student of this Association to carefully and thoroughly study a drug which seems, to me at least, so valuable, I shall feel that my humble remarks have been awarded much honor.

